# Civility vs. Incivility in Online Social Interactions: An Evolutionary Approach

**DOI:** 10.1371/journal.pone.0164286

**Published:** 2016-11-01

**Authors:** Angelo Antoci, Alexia Delfino, Fabio Paglieri, Fabrizio Panebianco, Fabio Sabatini

**Affiliations:** 1 Department of Economics and Management, University of Sassari, Sassari, Italy; 2 Department of Economics, London School of Economics and Political Science, London, United Kingdom; 3 National Research Council, Institute of Cognitive Sciences and Technologies, Rome, Italy; 4 Bocconi University and IGIER, Milan, Italy; 5 Department of Economics and Law, Sapienza University of Rome, Rome, Italy; Middlesex University, UNITED KINGDOM

## Abstract

Evidence is growing that forms of incivility–e.g. aggressive and disrespectful behaviors, harassment, hate speech and outrageous claims–are spreading in the population of social networking sites’ (SNS) users. Online social networks such as Facebook allow users to regularly interact with known and unknown others, who can behave either politely or rudely. This leads individuals not only to learn and adopt successful strategies for using the site, but also to condition their own behavior on that of others. Using a mean field approach, we define anevolutionary game framework to analyse the dynamics of civil and uncivil ways of interaction in online social networks and their consequences for collective welfare. Agents can choose to interact with others–politely or rudely–in SNS, or to opt out from online social networks to protect themselves from incivility. We find that, when the initial share of the population of polite users reaches a critical level, civility becomes generalized if its payoff increases more than that of incivility with the spreading of politeness in online interactions. Otherwise, the spreading of self-protective behaviors to cope with online incivility can lead the economyto non-socially optimal stationary states. **JEL Codes**: C61, C73, D85, O33, Z13. **PsycINFO Codes**: 2240, 2750.

## Introduction

There is growing evidence that “online incivility” is spreading across social networking sites (SNS) making them a potentially hostile environment for users ([1]–[3]). The definition of incivility has been long debated by communication scholars ([4]). In their study about televised incivility, [5] referred to it as the violation of well-established face-to-face social norms for the polite expression of opposing views. To the purpose of our study, we define online incivility as a manner of offensive interaction that can range from aggressive commenting in threads, incensed discussion and rude critiques, to outrageous claims, hate speech and harassment.

The Pew Research Center (PRC) has documented the rising incidence of incivility in SNS-based interactions: for example, 73% of online adults have seen someone being harassed in some way in SNS, and 40% have personally experienced it. 49% of SNS-using adults have seen other users behaving cruelly, 60% witnessed someone being called offensive names, and 53% had seen efforts to purposefully embarrass someone. 92% of Internet users agreed that SNS-mediated interaction allows people to be more rude and aggressive, compared with their offline experiences ([2]). The Facebook “Pages” and the Twitter accounts of actors of public interest such as political parties, magazines, and celebrities provide a typical setting for online incivility ([3]). In these settings, SNS users can randomly interact with strangers who subscribed to the same feed. Even if subscribers may have specific interests in common, they are likely to be heterogeneous in terms of personal traits, preferences, and modes of social interaction ([6]–[8]).

Interaction in SNS leads individuals to condition their behavior on the behavior of other users, in a strategic manner. For example, users may react to a hostile online environment where incivility is prevalent by in turn behaving rudely, or by abandoning the social network.

We study the evolution of online and offline social interaction in a mean field evolutionary game framework where individuals can choose whether to be polite or not when interacting with others in SNS. Everyone also has the option of opting out from SNS to cope with the possible hostility of the online environment.

We model a homogeneous population, where individuals have the same access to technologies, but can pursue three different strategies of social interaction: 1) using SNS and behaving politely in online interactions; 2) using SNS and behaving in an uncivil way in online interactions; 3) opting out from SNS. For the sake of simplicity, we assume that departing SNS users reduce their social participation to the minimum amount of face-to-face interactions that are inevitably required in everyday life (e.g. the line at the supermarket and the interaction with the cashier). This strategy can be interpreted as a form of self-protective behavior, which emerges when the combined hostility of the virtual social environments that surround the individual prompts a drastic form of adaptation consisting in the withdrawal from any significant (offline or online) interaction with others. We define the equilibrium in which all individuals choose social isolation as a “social poverty trap” ([9]).

The analysis of dynamics shows that the spreading of self-protective behaviors triggered by online incivility entails undesirable results to the extent to which it leads the economy to non-socially optimal stationary states that are Pareto dominated by others. For individuals, self-protective behaviors are rational in that they temporarily provide higher payoffs. However, their spreading causes a generalized decrease in the payoffs associated with each social participation strategy, which, in the long run, leads the economy to a non-optimal stationary state. The social poverty trap is always a locally attractive Nash equilibrium. When the other stationary states are attractive, they always give higher payoffs than the social poverty trap.

Our contribution bridges three literatures. The first literature is that of economists and political scientists who empirically analyzed how Internet use may impact on aspects of social capital such as face-to-face interactions and well-being (e.g. [10]–[13]). Our study contributes to this literature by introducing the problem of online incivility and providing the theoretical analysis of how the evolution of offline and online interactions can impact collective welfare.

Our focus on social poverty traps is also related to previous economic and sociological studies that analyzed how economic growth and technological progress may cause a decline in face-to-face social interactions ([14]–[15]), and to the literature concerning the “decline of community life thesis” ([16]).

The second body of literature comprises physicists and economists studying evolutionary games on networks, both theoretically and experimentally. Several authors have analyzed the topological structure of interactions in networks in an evolutionary game framework (starting from the seminal work of Nowak and May [17], a large literature grew. For a review see [18], [19] or [20]. For the specific contribution of economic thinking to this debate see, for example, [21]–[22]). We aim to add to this literature by building a mean field evolutionary framework to model the interactions that users regularly and randomly have with known and unknown others adopting different strategies of interaction in SNS. We also relate to the literature about voluntary participation, or opting-out, which proved to be a mechanisms fostering cooperation in networks (see, for example, [23]–[24]). In our case, instead of a complete opting out from the game, we model the possibility of a partial opting out from the sole SNS relationships.

The third body of literature is that of psychologists and computer scientists who have analyzed the impact of SNS use on social capital and well-being (e.g. [25]–[27]).

### The decline in social engagement

In his best seller *Bowling Alone*, Robert Putnam [14] documented that a decline in measures of social capital–such as participation in formal organisations, informal social connectedness, and interpersonal trust–began in the United States in the 1960s and 1970s, with a sharp acceleration in the 1980s and 1990s.

Putnam’s “decline of community life thesis” ([16]) prompted a number of subsequent empirical tests. [28] used a number of different sources to assess the development of social capital in the United States since 1952. The authors found a decline in indicators of volunteering, membership of organisations and entertainment with friends and relatives. Based on GSS data, [29] found a declining trend in indicators of social connectedness and confidence in institutions in the United States between 1975 and 2002.

Apart from the United States, there seems to be a common pattern of declining trust, social engagement and organisational activity across industrialised democracies starting from the 1980s, with the exception of Scandinavian countries ([30]). Declining trends of indicators of social interaction have been documented for England and Wales over the period 1972–99 ([31]), Great Britain over 1980–2000 ([32]), China ([33]) and Australia over 1960–90 ([34]).

Putnam [14] discussed three main explanations for the decline in American social capital: 1) the reduction in the time available for social interaction–related to the need to work more, to the rise in labour flexibility and to the increase in commuting time in urban areas; 2) the rise in mobility of workers and students; and 3) technology and mass media.

In the last decade, Putnam’s arguments have found support in a number of studies investigating the effect exerted on various dimensions of social connectedness by the rise in working time ([35]), labour mobility ([36]), urban sprawl and commuting ([37]), and the impoverishment of the social environment, which can prompt individuals to pursue isolation ([9]).

[9] modelled the decline in social engagement as the result of a self-protective reaction to the reduction in the time available for social activities, the decline in social participation opportunities and the rise of materialistic values. According to the authors, the need to “defend” oneself from an unfriendly environment where social engagement becomes increasingly less rewarding prompts the substitution of relational goods with private goods in individual preferences, thereby favouring social isolation. Social isolation can be interpreted as a particular form of self-defense through which individuals make their utility independent from the actions of others. For example, individuals choosing social isolation tend to watch a movie alone through a home theatre system instead of going to the cinema with friends. They may even prefer to renounce their leisure activities to devote all of the available time to work. In this way, their payoffs do not vary with the closing of theatres or with the decline in the number (or even the unavailability) of friends with whom to share a night at the cinema. This shift in preferences is not driven by mutating tastes. Rather, as explained by Hirsch [38], it must be interpreted as a self-protective reaction to the deterioration of the social environment. Hirsch was the first to introduce the concept of defensive consumption choices in his seminal work on the social limits to growth. This kind of consumption occurs in response to a change in the physical or social environment: “If the environment deteriorates, for example, through dirtier air or more crowded roads, then a shift in resources to counter these “bads” does not represent a change in consumer tastes but a response, on the basis of existing tastes, to a reduction in net welfare” ([38], p. 63).

### The rise in SNS-mediated interaction

In *Bowling Alone* [14], Putnam argued that progress in information technology could further exacerbate the decline in community life. At the time, Putnam referred to the negative role of television and other forms of technology-based entertainment such as video players and videogames. Early Internet studies reprised Putnam’s arguments suggesting that the Internet might displace even more social activities than television ([39]). The displacement hypothesis was supported by the first empirical tests of the relationship between Internet use and face-to-face interactions (e.g. [40]–[41]).

These explorations, however, were carried out before the rise of SNS, when using the Internet was predominantly a solitary activity with limited relational implications. Today, Internet use is closely related to engagement in online social networks (hereafter we will use the terms *social networking sites* and *online social network* as synonyms for the sake of brevity).

According to the Pew Research Center (PRC) Internet & American Life Project Survey, as of September 2014, 71 per cent of online adults were active on Facebook, 23 per cent used Twitter, 28 per cent used Pinterest and 26 per cent used Instagram ([42]).

These Figures mark a dramatic increase from 2009, when the PRC first began collecting data on Internet use. At that time, 46 per cent of online adults had ever used a SNS ([43]). Despite the extent of this transformation, the economic research on online networks is limited. In the fields of social psychology and communication science, several authors have tackled the potential role of SNS in face-to-face interaction in small samples of students in American colleges ([25]–[26], [44]).

In economics, a few studies empirically assessed the role of broadband on aspects of social capital and political participation but, due to a lack of data, they could not tackle the possible role of online social networks. Based on German Socio-Economic Panel data, [13] found that having broadband Internet access at home has positive effects on individuals’ social interactions, manifesting in a higher frequency of visiting theatres, opera and exhibitions, and in a higher frequency of visiting friends. Using data on Italian municipalities, [11] found that the diffusion of broadband led, initially, to a significant decline in electoral turnout in national parliamentary elections. This was reversed in the 2013 elections when the first round took place after the explosive rise of SNS. [10] found that the progressive increase in DSL availability significantly decreased voter turnout in German municipalities.

[45] theoretically analysed the evolution of social participation and the accumulation of social capital in relation to technological progress and online networking. Their results suggest that, under certain conditions, the stock of information and social ties accumulated within online networks can create an infrastructure that helps individuals to develop their social participation despite space and time constraints.

Overall, the evidence suggests that face-to-face and SNS-mediated interaction may be complementary, rather than one substituting the other. On the other hand, there is evidence that, despite the steep rise in the use of SNS, a decreasing yet still remarkable share of online adults chooses not to use or even to abandon them (see for example [46], for the U.S. and [47], for Italy).

### The problem of online incivility

The rise of SNS-mediated interaction has been accompanied by the emergence of new, unexpected, downsides. Anecdotal and descriptive evidence suggests that interaction in online social networks has increasingly been plagued by online incivility ([1],[2]). The roots of incivility in SNS-mediated social interactions have been addressed in a few psychological studies, which suggest that, when it comes to the presentation of opposing views and opinions, there is a fundamental difference between face-to-face and Internet-mediated interactions.

In contrast to online conversations, face-to-face interactions entail the use of expressions, smiles, eye contact, tone of voice, gesturing, and other nonverbal behavior that makes it easier to correctly perceive the interlocutors’ feelings and intentions. Online conversations, on the other hand, are more vulnerable to incomprehension and misunderstandings. In SNS-mediated interactions, interlocutors are basically ‘invisible’ and their feelings and sensitivities can hardly be perceived. As stated by [48] in an early study on computer-mediated communication, “Communicators must imagine their audience, for at a terminal it almost seems as though the computer itself is the audience. Messages are depersonalized, inviting stronger or more uninhibited text and more assertiveness in return”. [48] observed that computer-mediated communication entails anonymity, reduced self-regulation, and reduced self-awareness. “The overall weakening of self or normative regulation might be similar to what happens when people become less self-aware and submerged in a group, that is, *deindividuated*” (p. 1126). Deindividuation has in turn been found to be conducive to disinhibition and lack of restraint ([49]).

As a result, while in physical interactions people usually think twice before behaving offensively with a person who expresses an opposing view, SNS users are likely to care less about the risk of offending others in online conversations. In a pioneering experiment comparing face-to-face and online conversations, [50] found that people in computer-mediated groups were more aggressive than they were in face-to-face groups. In general, they were more responsive to immediate textual cues, more impulsive and assertive, and less bound by precedents set by societal norms of how to behave in groups. Based on survey data collected in a big U.S. company, [51] found that computer-mediated communication has substantial deregulating effects and encourages disinhibition in respect to non-mediated interactions.

Further studies suggested that a more impulsive and assertive behavior that does not consider the recipients’ feelings is far more common in Internet-mediated discussions as compared to face-to-face encounters ([52]). This phenomenon has been conceptualized as “flaming” ([50]). It refers to the expression of strong and uninhibited opinions, consisting of extreme emotional behavior expressed through uninhibited speech (insulting, offending, hostile comments, etc.).

The experimental studies mentioned above were conducted in very limited networks that were created ad hoc by researchers. It is reasonable to argue that in large networks such as Facebook and Twitter deindividuation and, therefore, disinhibition are likely to be exacerbated. Recent studies on Facebook have shown that controversial content was more frequent than any prosocial content categories, suggesting that there is an overrepresentation of negative content on the platform ([53]). A further distinctive element of interaction in big online networks is that possible reactions to provocative behaviours can be easily neutralized, for example by simply switching off the device, or even by ‘blocking’ the interlocutor through the network’s privacy settings. These ‘exit options’ probably further weaken inhibitions and self-regulation ([3]). By contrast, one cannot easily withdraw from an unpleasant face-to-face discussion.

The problem of incivility is important because the infringement of social norms for the polite expression of opposing views can provoke emotional and behavioral responses with relevant economic and political consequences. [5] experimentally analyzed the impact of incivility in mediated communication on trust. The authors noted a fundamental difference between face-to-face and television-mediated discussions about political views. Television-mediated presentations of opposing opinions often violate face-to-face social norms and easily deviate from civility. [5] collected experimental evidence that witnessing televised incivility causes a loss of trust in others. The authors claimed that, when social norms of politeness are violated in televised debates, watchers might feel hurt as if they personally experienced the offences they saw on TV. [54] argued that when incivility takes place in SNS-mediated interactions, users’ feelings might be affected as if the offences where perpetrated in real life. In respect to what may happen with televised incivility, witnessing online incivility entails a more intense emotional involvement not only because one can be personally targeted with offensive behaviors but also because when others are being offended in online environments there is a concrete possibility to intervene in their defense. Based on Italian survey data, [54] found that SNS users have significantly lower levels of trust in strangers, in neighbors, and in institutions than non-users and that such a decline in trust may be detrimental for users’ well-being. The use of SNS could cause a decline in trust through different mechanisms, some of which have already been mentioned: for instance, increased awareness of diversity, experience of new social norms and more frequent exposure to incivility as compared to face-to-face interactions. Overall, the evidence regarding online incivility suggests that SNS can easily become a hostile environment for users and prompts the need to analyze two different strategies of social interaction via SNS, based on the propensity for acting civilly or not.

## Model and Methods

We assume that agents can choose between two different ways of social involvement: 1) Active social involvement, entailing the development of interpersonal relationship both by means of face-to-face and SNS-mediated interactions. 2) Limited social involvement, entailing the opting out from SNS and the maintenance of the minimum amount of face-to-face interactions that are required for the completion of everyday life task, such as, for example, the interaction with the cashier at the supermarket and limited on-the-job interactions.

In addition, socially active agents who do not opt out from online social networks can also choose to behave either politely or rudely in SNS-mediated interactions.

In detail the strategies resulting from these possible choices are as follows:

1. *Strategy H*: social relations are developed both by means of face-to-face interactions and via social networking sites. SNS users who choose *H* (for Hate) behave online in an uncivil way. For example, these agents indulge in offensive and disrespectful behaviors, incensed discussions and rude critiques, outrageous claims and hate speech.
2. *Strategy P*: agents who follow this strategy develop their social relations both by means of face-to-face interactions and via SNS. In contrast to *H* players, however, *P* players behave politely in online interactions. We call this strategy *P* (for Polite).
3. *Strategy N*: agents following this strategy choose to withdraw from SNS-mediated relations and reduce face-to-face relations to the minimum. We label this strategy as *N* (for No SNS participation) and we call the equilibrium in which all individuals play *N* a “social poverty trap” ([9]). The withdrawal from SNS interactions modeled with the *N* strategy may be viewed as a drastic form of adaptation to the hostility of the environment that make *N* players’ payoff constant and completely independent from the behavior of others.

We first notice that the *N* strategy is a sort of opting out strategy. As discussed in the introduction, these kinds of strategy have been proved useful to foster cooperation in social dilemmas. However, contrary to standard opt out strategies, here agents playing *N* remain in the game but choose a “lower” level of interaction. When a *P* or a *H* agent switches to the *N* strategy, she breaks SNS connections with other people, while when an agent switches from *N* to *P* or *H* strategy, she creates SNS connections.

In each instant of time *t*, individuals interact with many others. The interactions between *H* and *P* individuals take place both face to face and via SNS. Those in which *N* agents are involved are reduced to the minimum face-to-face encounters needed.

### Payoffs

**Fig 1:**
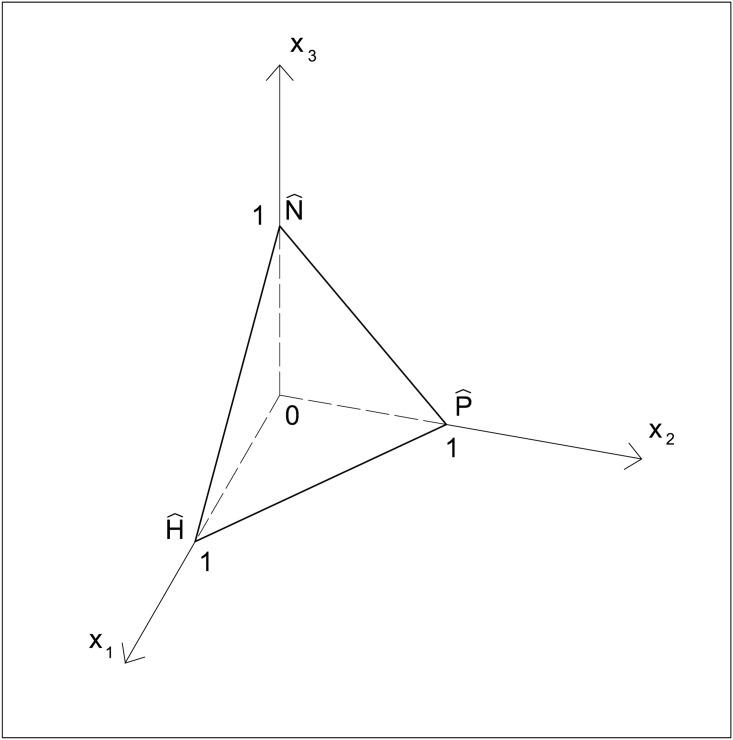
The two-dimensional simplex *S* in the space (*x*_1_, *x*_2_, *x*_3_). The points (*x*_1_, *x*_2_, *x*_3_) = (1, 0, 0), (*x*_1_, *x*_2_, *x*_3_) = (0, 1, 0), and (*x*_1_, *x*_2_, *x*_3_) = (0, 0, 1) correspond, respectively, to the vertices $\hat{H}$, $\hat{P}$, and $\hat{N}$, where all individuals play, respectively, strategies *H*, *P*, and *N*. Along the edge joining $\hat{H}$ and $\hat{P}$ (respectively, $\hat{H}$ and $\hat{N}$, $\hat{P}$ and $\hat{N}$) the strategy *N* (respectively, *P*, *H*) is not played.

Let us indicate by *x*_1_(*t*), *x*_2_(*t*) and *x*_3_(*t*) the shares of individuals adopting strategies *H*, *P*, and *N*, respectively, at time *t*. It holds *x*_*i*_(*t*) ≥ 0, all *i*, and $\sum_{i}x_{1}(t)=1$, therefore the vector *x*(*t*) = (*x*_1_(*t*), *x*_2_(*t*), *x*_3_(*t*)) belongs to the three-dimensional simplex *S* represented in Fig 1.

We assume that each agent interacts with agents of different types contemporarily. We call $x^{j}(t)=\left(x_{1}^{j}(t),x_{2}^{j}(t),x_{3}^{j}(t)\right)$ the vector of shares of the different strategies played by the sub-population of agents with whom the individual *j* is matched at each instant of time *t*. Then we assume that the payoff $\Pi_{i}^{j}$ that each agent *j* gets from playing strategy *i* depends on *x*^*j*^(*t*), $\Pi_{i}^{j}\left(x^{j}(t)\right)$.

Following standard mean field analysis, we assume that the types of agents an individual can meet in her social interactions reflects the average composition of the population. Thus, at each time *t*, each agent has neighbors of different types according to shares *x*_1_(*t*), *x*_2_(*t*), *x*_3_(*t*). This is a standard assumption in the economic analysis of complex networks (see, for example, [55]–[56]), which allows us to better focus on how changes in average population shares affect the payoff and the adoption dynamics of strategies. More specifically, we are interested in analyzing the adoption of the opting out strategy *N*, rather than in the effects that the specific composition of neighborhoods have. While the specific topological structure of the interactions plays a decisive role in shaping some features of the dynamics (see [18]–[20]), this simplification allows us to obtain a complete classification of the dynamics and to determine the average effects of the different population shares on the stationary state.

Consequently $\Pi_{i}^{j}\left(x^{j}(t)\right)=\Pi_{i}\left(x(t)\right)$, that is the payoff of playing a given strategy is independent of the specific identity of the player and just depends on the population shares and on the strategy played.

The strategic context of the game is illustrated by the following payoff matrix representing the payoffs of row-players when meeting a homogenous set of agents playing column strategy.

$$\begin{array}{cccc} & meets\;only\;H & meets\;only\;P & meets\;only\;N\\ H & \beta & \gamma & 0\\ P & -\delta & \varepsilon & 0\\ N & \eta & \eta & \eta \end{array}$$

When a row player meets a heterogeneous set of agents, then her payoffs are derived by the convex linear combination of payoffs whose weights are the population shares. Consider first *H* and *P* strategies:

$$\Pi_{H}\left(x_{1},x_{2}\right)=\beta x_{1}+\gamma x_{2}$$

$$\Pi_{P}\left(x_{1},x_{2}\right)=-\delta x_{1}+\varepsilon x_{2}$$

Notice that, with this specification, the payoffs do not depend on the number of interactions each agent has (and thus on the degree of each agent in the network), but on the shares of strategies in own neighborhood.

The payoff of the *N* strategy is assumed to be constant and, therefore, it does not depend on the distribution (*x*_1_, *x*_2_, *x*_3_) of strategies:

$$\Pi_{N}=\eta$$

We assume *δ*, *ε*, *η* > 0. The strict positivity of *η* characterizes *N* as a self-protective strategy: in a context where no one engages in social interactions, *N* becomes the best performing strategy. We also assume that the payoff from virtuous social interactions (i.e. adopting strategy *P*) is increasing in the proportion of people interacting in such a way (*ε* is positive). Finally, we assume the impact of the diffusion of the “hate” strategy on a polite’s payoff is always negative (*δ* is positive).

We instead allow the parameters *β* and *γ* to be either positive or negative. It is not clear, in fact, whether haters get more satisfaction when dealing with polite SNS users or by confronting with others of the same type. An *H* player, for example, may find the interaction with a polite player who defuses provocations with kindness less rewarding; accordingly, we allow *H* players to get disutility from the interaction with a polite person. Or, by contrast, she may find it harder, and less rewarding, to confront another hater.

Notice that:

1. The population state $\hat{N}=\left(x_{1},x_{2},x_{3}\right)=(0,0,1)$ –where all individuals play the *N* strategy–is always a (strict) Nash equilibrium.
2. The population state $\hat{H}=\left(x_{1},x_{2},x_{3}\right)=(1,0,0)$ –where all individuals play the *H* strategy–is a Nash equilibrium if and only if *β* > *η*.
3. The population state $\hat{P}=\left(x_{1},x_{2},x_{3}\right)=(0,1,0)$ –where all individuals play the *P* strategy–is a Nash equilibrium if and only if *ε* > *γ*, *η*.
4. The pure population states $\hat{N}$, $\hat{H}$, and $\hat{P}$ can simultaneously be Nash equilibria.
5. The payoff of each individual in the state $\hat{N}$ (given by *η*) is lower than the payoff of each individual in the states $\hat{H}$, and $\hat{P}$ (given, respectively, by *β*, and *ε*) when such states are Nash equilibria.
6. The *N* strategy is never dominated by the other strategies. The *H* strategy is dominated by *N* if *η* ≥ max (*β*, *γ*), while it is dominated by *P* if *β* ≤ −*δ* and *γ* ≤ *ε*. Finally, the *P* strategy is dominated by *N* if *ε* ≤ *η*, while it is dominated by *H* if *β* ≥ −*δ* and *γ* ≥ *ε*.

According to a well-known result in evolutionary game theory (see, e.g., [57]), if the pure population states $\hat{N}$, $\hat{H}$, and $\hat{P}$ are Nash equilibria, then they also are (locally) attractive stationary states under every payoff-monotonic adoption dynamics of strategies. Consequently, in the contexts in which $\hat{N}$ is not the unique existing Nash equilibrium, the adoption dynamics are path dependent in that different stationary states may be reached starting from different initial distributions (*x*_1_(0), *x*_2_(0), *x*_3_(0)) of strategies.

The stationary state $\hat{N}$ can be interpreted as a *social poverty trap*, in the sense of [9]; that is, as an attractive stationary state where aggregate social participation and welfare (measured by payoffs) fall to the lowest possible level with respect to other stationary states.

To focus our analysis on more relevant cases only, we shall study adoption dynamics under the assumption that no strategy is dominated by others (see Point 6 above). Such assumption requires the following restrictions on parameters’ values:

ε>η, max(β,γ)>η, (1)

either β>−δ and γ<ε or β<−δ and γ>ε (2)

Notice that:

1. In the context in which *β* > −*δ* and *γ* > *ε* hold (see Eq (2)), also the state $\hat{P}=\left(x_{1},x_{2},x_{3}\right)=(0,1,0)$ –where all individuals play the *P* strategy–is a Nash equilibrium (and, therefore, a locally attractive stationary state), while the state $\hat{H}=\left(x_{1},x_{2},x_{3}\right)=(1,0,0)$ –where all individuals play the *H* strategy–may be a Nash equilibrium (this is the case only if *β* > *η*) or not.
2. In the context in which *β* < −*δ* and *γ* > *ε* hold (see Eq (2)), the states $\hat{P}$ and $\hat{H}$ are never Nash equilibria (and, therefore, they are never attractive). As we will see, such a context favours the coexistence of the *H* and the *P* strategy. Importantly, this context captures an interesting set of social scenarios: the first condition, *β* < −*δ*, requires that a *H* player is more negatively affected by interacting with *H* players than what would happen to a *P* player, suggesting that (i) haters do not get along with each other, possibly because they get no satisfaction in the absence of a proper “victim” and/or are forced to take a taste of their own medicine, whereas (ii) polite people are only mildly annoyed by interacting with haters. On the other hand, the second condition, *γ* > *ε*, implies that interacting with *P* players is more satisfactory for a *H* player than for another *P* player.

### Evolutionary dynamics

Following [58], we assume that the diffusion of the three strategies is described by the replicator equations:

$${\dot{x}}_{1}=x_{1}\left[\Pi_{H}\left(x_{1},x_{2}\right)-\bar{\Pi }\left(x_{1},x_{2},x_{3}\right)\right]$$ (3a)

$${\dot{x}}_{2}=x_{2}\left[\Pi_{P}\left(x_{1},x_{2}\right)-\bar{\Pi }\left(x_{1},x_{2},x_{3}\right)\right]$$ (3b)

$${\dot{x}}_{3}=x_{3}\left[\Pi_{N}-\bar{\Pi }\left(x_{1},x_{2},x_{3}\right)\right]$$ (3c)

Where ${\dot{x}}_{1}$, ${\dot{x}}_{2}$, and ${\dot{x}}_{3}$ represent the time derivatives of the functions *x*_1_(*t*), *x*_2_(*t*), and *x*_3_(*t*), respectively, and:

$$\bar{\Pi }=x_{1}\Pi_{H}+x_{2}\Pi_{P}+x_{3}\Pi_{N}$$

is the population-wide average payoff of strategies.

Dynamics Eqs (3a, 3b and 3c) are defined in the simplex *S* illustrated in Fig 1, where *x*_1_, *x*_2_, *x*_3_ ≥ 0 and *x*_1_ + *x*_2_ + *x*_3_ = 1 hold.

According to replicator Eqs (3a, 3b and 3c), individuals tend to imitate players who adopt the relatively more rewarding strategies. As a consequence, such strategies spread in the population at the expenses of the less rewarding ones.

All the pure population states $\hat{N}$, $\hat{H}$, and $\hat{P}$ are stationary states under dynamics Eqs (3a, 3b and 3c). Furthermore, the edges of *S* where one strategy is adopted by no one are invariant under dynamics Eqs (3a, 3b and 3c); that is, every trajectory starting from a point belonging to one of the edges, remains in the edge for every time *t* ∈ (−∞, +∞). Replicator dynamics, and any other payoff-monotonic dynamics, represent *selection*, as opposed to *mutation*, in the sense that they represent the selection process of *present* behaviors (pure strategies) via imitation of the more rewarding ones, while *absent* behaviors remain absent ([57], p. 140). When a new strategy enters the “market”, it can be adopted (imitated) by agents only if, at the initial time *t* = 0, a strictly positive share of agents decide to adopt it. Initial strategy choices *x*_1_(0), *x*_2_(0), and *x*_3_(0) are considered as exogenously determined and may be influenced by several factors.

## Results

### Classification of dynamic regimes

The analysis of systems (3a, 3b and 3c) builds on the classification results in Bomze [59]. It allows us to give a complete classification of all the possible dynamic regimes that may be observed under systems (3a, 3b and 3c). The computations allowing us to apply Bomze’s classification to our model are very simple and we omit them (see [45], for an example of application of Bomze’s classification method).

**Fig 2:**
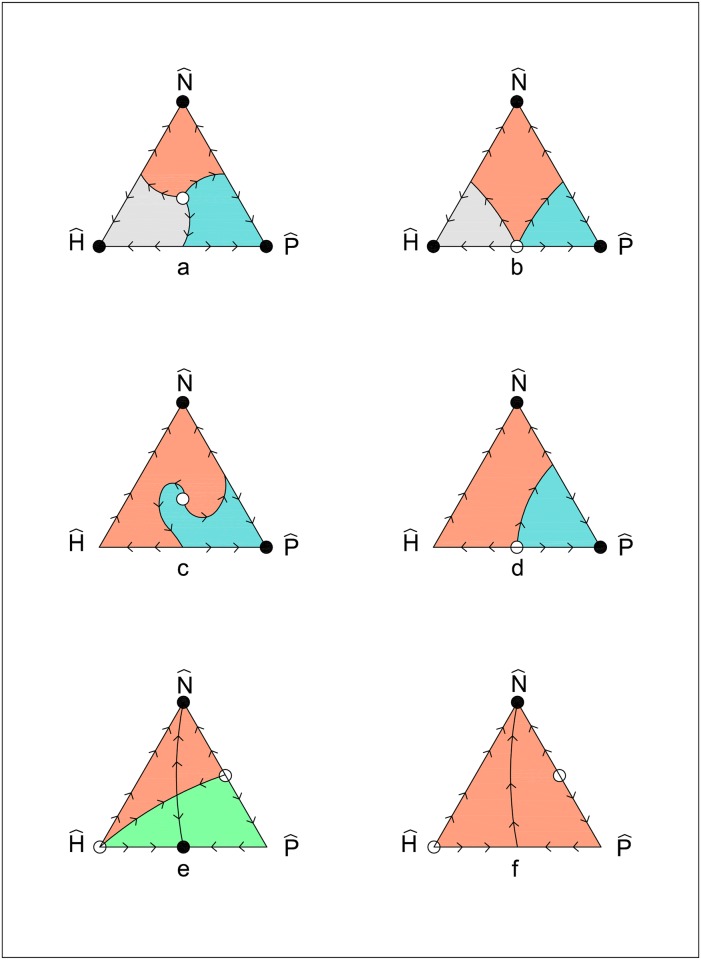
The taxonomy of dynamic regimes. In these figures, a full dot • represents a locally attractive stationary state, an empty dot ∘ represents a repulsive stationary state, while a saddle point is indicated by drawing its insets and outsets. Only some representative trajectories are sketched.

The dynamic regimes that can be observed are illustrated in Fig 2a–2f. In these figures, a full dot • represents a locally attractive stationary state, an empty dot ∘ represents a repulsive stationary state, while a saddle point is indicated by drawing its insets and outsets (stable and unstable manifolds, respectively). Only some representative trajectories are sketched. For simplicity, in this classification, we omit consideration of non-robust dynamic regimes, that is, the regimes observed only if an equality condition on parameters' values holds. The dynamic regimes that may be observed in the simplex *S* are the following:

**Case 1**: *γ* > *ε* (and therefore, by assumption Eq (2), *β* > −*δ*). We have two sub-cases:
  **1.a)** If *β* > *η*, then all the stationary states $\hat{N}$, $\hat{H}$, and $\hat{P}$ are locally attractive. No other attractive stationary state exists. Fig 2a and 2b illustrate the corresponding dynamic regimes. They correspond, respectively, to phase portraits number 7 and 35 in Bomze’s classification (from now on, we shall indicate the *phase portrait number # of Bomze’s classification* with the symbol *PP#*).
    The regime in Fig 2a is observed if the condition (*ε* − *γ*)(*η* − *β*) + (*β* + *δ*)(*η* − *γ*) < 0, that is:
    $$\eta <\frac{\beta \varepsilon +\gamma \delta }{\varepsilon -\gamma +\beta +\delta }$$ (4)
    holds. The regime in Fig 2b is observed if the opposite of condition (4) holds.
  **1.b)** If *β* < *η*, then the stationary states $\hat{P}$ and $\hat{N}$ are locally attractive, while $\hat{H}$ is a saddle point. Fig 2c (respectively, Fig 2d) illustrates the dynamic regime occurring if condition (4) (respectively, the opposite of Eq (4)) holds. Fig 2c and 2d correspond, respectively, to *PP*9 and *PP*37.
**Case 2:** *γ* > *ε* (and therefore, by assumption Eq (2), *β* < −*δ*). In this case, *β* < 0 holds and, therefore, *β* < *η*. According to these conditions on parameters, the stationary state $\hat{N}$ is locally attractive, $\hat{H}$ is repulsive and $\hat{P}$ is a saddle point. Furthermore, there exists another locally attractive stationary state:
  $$\left(x_{1},x_{2},x_{3}\right)=\left(x_{1}^{*},1-x_{1}^{*},0\right),\;x_{1}^{*}=\frac{\varepsilon -\gamma }{\varepsilon -\gamma +\beta +\delta }$$ (5)
  if condition (4) holds. The stationary state Eq (5) lies in the edge of the simplex where only strategies *H* and *P* are played (Fig 2f, which corresponds to *PP*11). If the opposite of condition (4) holds, then $\hat{N}$ is the unique attractive stationary state and the dynamic regime is that illustrated in Fig 2g (corresponding to *PP*36).

Notice that, in Case 2 (*γ* > *ε* and *β* < −*δ*), *ε* − *γ* + *β* + *δ* < 0 holds and therefore the right side of Eq (4) is positive if:

βε+γδ<0 (6)

To ease interpretation, condition (6) can be also expressed as *γ* / *β* > −*ε* / *δ*. This inequality compares the ratio of marginal return when meeting a polite user or a hater for haters versus polite users (abusing terminology, as a sort of *marginal rate of substitution* of haters vs. polites). If Eq (6) holds, at the margin the rate at which haters are willing to forgo meeting one hater for meeting a polite user is greater than the rate for polite users.

### Welfare

Individuals’ payoffs in the pure population stationary states $\hat{N}$, $\hat{H}$ and $\hat{P}$ are given respectively by Π_*P*_ = *η*, Π_*H*_(1, 0) = *β* and Π_*P*_(0, 1) = *ε*; furthermore $\hat{H}$ and $\hat{P}$ are attractive if, respectively, *β* > *η* and *ε* > *γ*, *η* hold. Consequently, individuals’ welfare in $\hat{H}$ and $\hat{P}$ is higher than in $\hat{N}$, when $\hat{H}$ and $\hat{P}$ are attractive. However, the stability conditions concerning $\hat{H}$ and $\hat{P}$ do not allow us to order them in terms of welfare.

In addition, in the stationary state $\left(x_{1},x_{2},x_{3}\right)=\left(x_{1}^{*},1-x_{1}^{*},0\right)$, where only the strategies *H* and *P* are played (see Eq (5)), individuals’ payoff is given by:

$$\Pi_{H}\left(x_{1}^{*},1-x_{1}^{*}\right)=\Pi_{P}\left(x_{1}^{*},1-x_{1}^{*}\right)=\frac{\beta \varepsilon +\gamma \delta }{\varepsilon -\gamma +\beta +\delta }$$ (7)

It is easy to check that:

$$\varepsilon =\Pi_{P}\left(0,1\right)>\Pi_{H}\left(x_{1}^{*},1-x_{1}^{*}\right)=\Pi_{P}\left(x_{1}^{*},1-x_{1}^{*}\right)>\Pi_{H}\left(1,0\right)=\beta$$

holds: individuals’ welfare in $\left(x_{1}^{*},1-x_{1}^{*},0\right)$ is lower than in the stationary state $\hat{P}$ and higher than in the stationary state $\hat{H}$. Notice that condition (4) holds if and only if (see Eq (7)):

$$\Pi_{H}\left(x_{1}^{*},1-x_{1}^{*}\right)=\Pi_{P}\left(x_{1}^{*},1-x_{1}^{*}\right)>\Pi_{N}=\eta$$

This implies that when the stationary state $\left(x_{1}^{*},1-x_{1}^{*},0\right)$ is attractive (this happens in Case 2, when condition (4) is satisfied), then individuals’ welfare in $\left(x_{1}^{*},1-x_{1}^{*},0\right)$ is higher than in the social poverty trap $\hat{N}$.

## Discussion

In this paper we have built an evolutionary game model to study social interaction in a society where social environment can become hostile due to the increase in the share of the population adopting the self-protective *N* strategy (entailing the choice of social isolation) and the impolite *H* strategy (entailing the adoption of an uncivil behavior in online interactions).

Our analysis showed that the three pure population stationary states $\hat{N}$, $\hat{H}$, and $\hat{P}$, where all individuals adopt the same strategy (respectively *N*, *H*, and *P*), can be simultaneously attractive only if *β* > −*δ* and *γ* > *ε* hold. In such a context, no attractive stationary state in which two or three strategies are played exist. The coexistence between strategies can only be observed when *β* < −*δ* and *γ* > *ε*. In such a context, an attractive stationary state $\left(x_{1},x_{2},x_{3}\right)=\left(x_{1}^{*},1-x_{1}^{*},0\right)$, where only the strategies *H* and *P* are played, can exist. The first condition, *β* < −*δ*, requires that a *H* player is more negatively affected by interacting with other *H* players than what would happen to a *P* player, suggesting that (i) haters do not get along with each other, possibly because they get no satisfaction in the absence of proper “victims” and/or are forced to take a taste of their own medicine, whereas (ii) polite people are only mildly annoyed by interacting with haters. On the other hand, the second condition, *γ* > *ε*, implies that interacting with *P* players is more satisfactory for a *H* player than for a *P* player. Our results suggest that politeness can survive in a world with a fair share of haters only if the payoffs of polite people are not heavily affected by haters. Thus it would seem that Internet users engaged with haters need to heed the same advice Virgil gave Dante upon entering Hell: “Let us not speak of them, but do thou look and pass on”.

Whatever the parameter configuration is, the stationary state $\hat{N}$ is always locally attractive, while those entailing positive levels of participation (that is, the stationary states $\hat{H}$, $\hat{P}$ and $\left(x_{1}^{*},1-x_{1}^{*},0\right)$) can be attractive or not, according to the configuration of parameters. When this happens, they always give higher payoffs than the social poverty trap $\hat{N}$.

The destination of dynamics strictly depends on the initial distribution of the strategies in the population. This path dependence suggests that societies that are similar along a number of fundamental features can converge to different equilibria depending on their initial distribution of strategies (*x*_1_(0), *x*_2_(0), *x*_3_(0)). Policies aimed at modifying individual payoffs might not be sufficient to prevent social poverty traps. From an institutional perspective, what could policy makers do to help people out of complete isolation and restore social interactions? Should governments intervene, or are there market forces that could be leveraged to do so? [9] extensively argue for the need for complementary actions between governments and civil society. However, this model is pessimistic about the role of civil society; when a social trap forms, the whole population converges to the pure strategy equilibrium $\hat{N}$, without any convenient individual deviation. The dissemination of information on the existence of incivility online and the reasons why it can be a serious problem for society should be of primary concern for policy makers, SNS managers and users alike. Therefore the government should probably enforce policies to prevent defensive self-isolating behaviors (e.g., school education on SNS and how to react to incivility) or to re-establish social connections (e.g., free public events, public goods with a social component). Future research should shed light on these issues.

In addition, future research might consider relaxing the mean-field assumption we adopted in our framework. In our model, the interaction between the various types of player mostly happens randomly. However, socialization is often driven by the tendency of individuals to associate and bond with similar others. While homophily commonly concerns socio-demographic characteristics, opinions and interests (see, for example, [60]–[61]), the strategies of online interaction we consider in this paper only focus on the personality traits determining whether an individual will behave politely or rudely on SNS–whatever her socio-demographic characteristics, opinions and interests are. This assumption can be justified by the fact that we do not model interactions in friendship networks, where homophily plays a crucial role, but we model random face-to-face daily interactions and interactions in SNS. These last ones involve friends, friends of friends and a large amount of agents with whom any SNS user randomly interacts. In our stylized framework, even assuming homophily to play a role, this would likely happen along the dimensions of gender, ethnicity, preferences and tastes, instead of the dimensions described by our strategies, which depend on deeper personality traits that are likely to be orthogonal to the drivers of homophily. Future research should address the role of homophily by analysing how the *P* and the *H* strategies interact with other users’ personal features such as, for example, their opinions. Uncivil behavior, in fact, is more likely to occur among individuals with different preferences or tastes. Homophily along specific preferences (concerning, for example, political issues) could impact the diffusion of the Polite and the Hate strategies in the population by encouraging the formation of segregated communities in which the Polite strategy could be much more profitable. This form of segregation could decrease online networks’ potential to contribute to the diffusion of knowledge and information.
